# Oral Supplementation with Sucrosomial Ferric Pyrophosphate Plus L-Ascorbic Acid to Ameliorate the Martial Status: A Randomized Controlled Trial

**DOI:** 10.3390/nu12020386

**Published:** 2020-01-31

**Authors:** Matteo Briguglio, Silvana Hrelia, Marco Malaguti, Elena De Vecchi, Giovanni Lombardi, Giuseppe Banfi, Patrizia Riso, Marisa Porrini, Sergio Romagnoli, Fabio Pino, Tiziano Crespi, Paolo Perazzo

**Affiliations:** 1 Scientific Direction, IRCCS Orthopedic Institute Galeazzi, Via Riccardo Galeazzi 4, 20161 Milan, Italy; banfi.giuseppe@fondazionesanraffaele.it; 2 Department for Life Quality Studies, University of Bologna, Corso d’Augusto 237, 47921 Rimini, Italy; silvana.hrelia@unibo.it (S.H.); marco.malaguti@unibo.it (M.M.); 3 Laboratory of Clinical Chemistry and Microbiology, IRCCS Orthopedic Institute Galeazzi, Via Riccardo Galeazzi 4, 20161 Milan, Italy; elena.devecchi@grupposandonato.it; 4 Laboratory of Experimental Biochemistry and Molecular Biology, IRCCS Orthopedic Institute Galeazzi, Via Riccardo Galeazzi 4, 20161 Milan, Italy; giovanni.lombardi@grupposandonato.it; 5 Department of Athletics, Strength and Conditioning, Poznań University of Physical Education, Królowej Jadwigi 27/39, 61-871 Poznań, Poland; 6 Faculty of Medicine and Surgery, Vita-Salute San Raffaele University, Via Olgettina 58, 20132 Milan, Italy; 7 Division of Human Nutrition, Department of Food, Environmental and Nutritional Sciences (DeFENS), University of Milan, Via Mangiagalli 25, 20133 Milan, Italy; patrizia.riso@unimi.it (P.R.); marisa.porrini@unimi.it (M.P.); 8 Joint Replacement Department, IRCCS Orthopedic Institute Galeazzi, Via Riccardo Galeazzi 4, 20161 Milan, Italy; sergio.romagnoli@libero.it; 9 Post-operative Intensive Care Unit & Anesthesia, IRCCS Orthopedic Institute Galeazzi, Via Riccardo Galeazzi 4, 20161 Milan, Italy; fabio_pino86@hotmail.it (F.P.); dott_crespi@hotmail.it (T.C.); paoloperazzo1@virgilio.it (P.P.)

**Keywords:** iron, anemia, vitamin, dietary supplements, nutraceutical, functional food, orthopedics, musculoskeletal diseases, older adult, frail, integrative medicine

## Abstract

Altered martial indices before orthopedic surgery are associated with higher rates of complications and greatly affect the patient’s functional ability. Oral supplements can optimize the preoperative martial status, with clinical efficacy and the patient’s tolerability being highly dependent on the pharmaceutical formula. Patients undergoing elective hip/knee arthroplasty were randomized to be supplemented with a 30-day oral therapy of sucrosomial ferric pyrophosphate plus L-ascorbic acid. The tolerability was 2.7% among treated patients. Adjustments for confounding factors, such as iron absorption influencers, showed a relevant response limited to older patients (≥ 65 years old), whose uncharacterized Hb loss was averted upon treatment with iron formula. Older patients with no support lost −2.8 ± 5.1%, while the intervention group gained +0.7 ± 4.6% of circulating hemoglobin from baseline (*p* = 0.019). Gastrointestinal diseases, medications, and possible dietary factors could affect the efficacy of iron supplements. Future opportunities may consider to couple ferric pyrophosphate with other nutrients, to pay attention in avoiding absorption disruptors, or to implement interventions to obtain an earlier martial status optimization at the population level.

## 1. Introduction

### Martial Status in Orthopedic Surgery 

In Italy, blood iron is understood to be part of the patient’s martial status (from Italian *profilo marziale*: *profilo* “profile” and *marziale* “martial”), which is primarily represented by circulating levels of hemoglobin (Hb), red blood cells (RBCs), transferrin (Tf), and ferritin. Blood values of Hb and/or RBCs below the normal ranges refer to the unspecific condition named “anemia” (from Greek *ἀναιμία*: *ἀν-* “without” and *-αἷμα* “blood”). Anemia is a biochemical sign that can mirror the dysfunction of hematopoietic organs, but might be also associated to hereditary diseases (e.g., sickle cell disease) or secondary conditions (e.g., vitamin B12 deficiency in pernicious anemia). The accurate diagnosis of anemia type should consider diverse blood indices, such as vitamins and inflammatory markers, but the criterion of Hb < 13 g/dL (♂) or < 12 g/dL (♀) always represents an alarm bell [1,2]. In Orthopedics, about 17.5% of all subjects undergoing elective hip or knee replacement present iron-deficiency anemia [3], 20%–30% have uncharacterized anemia [3,4], and over 50% may require preoperative blood transfusions [5], with trauma patients possibly presenting higher rates. Regardless of etiology, anemia conditions are associated with wound complications, readmissions, higher transfusions, and mortality rates [3,6,7,8,9]. The lower cytochrome activity grounds altered bactericidal activity of leucocytes [10,11,12,13] and can impair the patient’s immune response. Perioperative blood losses further compromise oxygen supply, thus creating an imbalance for increased demands that reduces cardiac ejection function [14], increases the risk of ischemia [15], lengthens skeletal muscles recovery, and impacts on breathing capacity—in turn, worsening the anesthesia-derived hypoventilation [16]. The required allogeneic blood transfusions expose institutions to higher costs, thus making it necessary to develop strategies to mitigate risks [17].

In 2015, the Patient Blood Management (PBM) program was introduced in Italy, with the scope of ensuring the appropriateness in the management of blood resources. The first pillar of this approach comprises the preoperative optimization of hematopoiesis [18]. In 2019, we proposed the integration of the hospital standard of care in Orthopedics with a Preoperative Optimization Clinic (POC) [9]. In this health facility, patients enlisted for major orthopedic surgery could undergo a program for correcting anemia conditions, thus ameliorating outcomes. Nowadays, an opportunity for anemia correction could be exploited during the anesthesia evaluation 30 days prior to surgery, when clinical and surgical needs are considered [19]. Most anemia types can be corrected through parenteral iron that bypasses the step of intestinal absorption, thus assuring the repletion of body storages often upon one-time administration. Nevertheless, this procedure is not without risk and requires specialized personnel with a dedicated ambulatory where patients can be monitored. An option for martial status optimization could be the use of oral iron supplements [20,21,22,23]. Different formulas have been available since decades, with iron salts (e.g., ferrous sulphate [24] and ferric pyrophosphate [25]) being widely used. Different pharmaceuticals may be preferred according to the specific setting of use, such as polysaccharide-iron complexes (e.g., ferric polymaltose [26]) for pregnant women or even microspheres of pure iron (i.e., carbonyl iron) for food fortification. However, gastrointestinal side effects were often reported, together with poor absorption and intestinal inflammation [27]. To avoid these issues, a novel formula consists of ferric pyrophosphate that is surrounded by a sucrosomial matrix made of phospholipids and sucrester: within this vehicle, iron absorption is maximized and the metal never gets in contact with mucosal cells, thus greatly increasing gastrointestinal tolerability. Nonetheless, blood tests are currently still questionable in identifying patients who should be preoperatively optimized, and false-positive results might increase hospital visits and augment patient’s anxiety [28]. Therefore, a population medicine approach may be more practical. Following PBM Italian indications and previous literature evidences, we planned a preoperative intervention to observe the efficacy and tolerability of an oral iron therapy in ameliorating the martial status of patients undergoing hip or knee surgery at IRCCS Orthopedic Institute Galeazzi, this intervention being one of the very first steps in building the future POC [9,29].

## 2. Materials and Methods

### 2.1. Study Design and Participants

The study was conducted at IRCCS Orthopedic Institute Galeazzi of Milan (Italy). The research was planned as an open-label randomized controlled trial on humans with no placebo, to study the efficacy of a dietary supplement of sucrosomial ferric pyrophosphate plus L-ascorbic acid in ameliorating the martial status of patients undergoing hip or knee prosthetic surgery. The recruitment period was set between January 2018 and June 2019, with screening and enrolment being conducted among consecutive patients referring to a single surgical unit during the anesthesia evaluation 30 days prior to surgery. A modification of Hb before and after treatment was set as primary outcome variable (efficacy), while the dietary supplement tolerability was set as secondary outcome (safety). A sample size of 82 patients comprising drop estimation was calculated to obtain a significant primary outcome (+ 0.80 g/dL of Hb after treatment). We screened patients who were candidates for first elective total hip or knee replacement with at least 1 months’ notice. Eligibility criteria were Caucasian race, years of age between 18–80, male or female genders, and a predicted operative risk score of the American Society of Anesthesiologists (ASA) ≤ 3. Exclusion criteria were neuropsychiatric disorders, orthopedic revisions, use of supplements, adverse reactions to supplements, iron disorders, myelo- or lympho-proliferative diseases, chronic kidney disease, tumors. On the same day of signing of the informed consent, patients were randomized by using an online tool that gives a random assignment (https://random.org/) between two groups: the control group followed the hospital standard of care with no placebo, whereas the dietary supplement was prescribed to the intervention group. According to the study protocol, about 30 days had to pass between the randomization and the day of surgery. Blood samples were collected after the informed consent signature (baseline, T0) and the day before surgery (T1). Patients were also monitored for complications a few days in the hospital ward (G0, G1, and G2). The San Raffaele Ethics Committee of Milan (Italy) approved and authorized the protocol (code: 158/int/2017) on February 8^th^ 2017, in compliance with the current Italian and international regulations governing the involvement of humans in clinical trials. The study was registered on a dedicated website (https://clinicaltrials.gov/) that assigned the code NCT04078880. The dataset with all demographic, clinical, and laboratory data that have been reported in this paper will be provided during the review process.

### 2.2. Intervention and Biochemical Analyses

The intervention group was supplemented daily with 1 capsule of 30 mg of iron plus 70 mg of L-ascorbic acid. Metallic taste, nausea, gastric distress, and dark stools are symptoms that are supposed to be avoided by using this pharmaceutical formula. For comprehensive insights into the pharmaceutical formula, see Appendix A. To monitor the martial status, laboratory tests were decided on the bases of logistics. Whole blood was collected before surgery at T0 (−30 days) and at T1 (day before/of surgery) and was processed within 3 hours from withdrawals on fully-automated analyzers. Ethylenediaminetetraacetic acid (EDTA) tubes (Becton Dickinson, Italy) and Sysmex XN (Dasit, Italy) were used for blood count and differential, which comprised Hb, RBCs (net count of erythrocytes), mean corpuscular volume (MCV, average size of a single erythrocyte), mean corpuscular hemoglobin (MCH, average amount of Hb inside a single erythrocyte), and mean corpuscular hemoglobin concentration (MCHC, average concentration of Hb inside a single erythrocyte). Blood samples in clot activator tubes (Becton Dickinson, Italy) were centrifuged (2600*g* at 4 °C for 15 min.). Serum iron (chromogen Ferene), Tf (immunoturbidimetric assay), Tf saturation (sat), and ferritin (chemiluminescence immunoassay) were measured on Architect ci 8200 analyser (Abbott, Italy). Tf is the transporter of iron and Tf sat is calculated as the percentage of binding sites on all Tf molecules occupied with iron. Ferritin is the serum protein that delivers iron to cells and reflects the amount of stored iron. See Appendix B for detailed descriptions and significances of blood indices.

### 2.3. Statistical Analyses

Adherence to the dietary supplement was strictly monitored and patients were asked to bring back empty boxes at T1. For this reason, data have been analyzed as originally allocated after randomization (intention-to-treat analysis). The Shapiro–Wilk test has been applied for distinguishing between normally distributed and skewed values. The continuous variables of years of age, MCV, MCH, iron, Tf, Tf sat, and ferritin have been analyzed as skewed, whereas Hb, RBCs, and MCHC have been analyzed as normally distributed values. All tests have been performed by using SPSS 22 and 2-tailed tests. Data between groups have been compared at T0 by using the independent sample *t*-test for normally distributed values or the Mann–Whitney U test for skewed values in order to state the absence of differences between groups, thus verifying the success of randomization. To investigate the efficacy of the dietary supplement, red blood cell indices and iron tests at T1 have been confronted to observe any group difference, which might reflect the efficacy of the dietary supplement in modifying the martial status of recruited patients. Unadjusted data have been compared by using the independent sample *t*-test or the Mann–Whitney U test. In order to identify if demographic variables could confound the response to treatment, first a Pearson’s correlation and then a linear regression analysis has been used to investigate the existence, direction, and strength of the association with our primary outcome variable. Paired samples *t*-test has been used to verify the treatment effects on Hb in subgroups of patients adjusted for randomization and specific variables. To investigate the safety of the dietary supplement, the occurrence of adverse effects associated with oral iron was monitored few days after surgery and reported as a rate of events comprising nausea, vomiting, gastric pain, constipation or diarrhea onsets.

## 3. Results

A total of 82 patients undergoing elective hip or knee replacement were selected and recruited, with nine patients counted as drop outs at trial completion. Baseline RBCs indices and iron profiles have been reported in Table 1.

Reasons for the drops were the following: two postponements of surgery, one unreliable to treatment, five missing blood withdrawals, one experiencing gastric distress to treatment (tolerability outcome). According to the World Health Organization (WHO) criterion (Hb cut-offs of 12–13 g/dL), about 8.2% of our patients suffered from anemia at baseline. 20.5% of all subjects were anemic with the Hb cut-off of 13 g/dL for both genders, and 6.8% according to our Hb laboratory ranges (♂: 13.7−17.5, ♀: 11.2−15.7). The mean age of the patients was 67.3 ± 8.6 years (range: 49−99) and the study involved 30 males and 43 females, grouped into 28 adults (< 65 years old) and 45 older adults (≥ 65 years old). The accuracy of randomization has been evaluated by confronting baseline (T0) data between the intervention and control groups: no significant differences have been observed for age (*p* = 0.356), Hb (*p* = 0.403), RBCs (*p* = 0.483), MCV (*p* = 0.608), MCH (*p* = 0.800), MCHC (*p* = 0.904), iron (*p* = 0.476), Tf (*p* = 0.402), Tf sat (*p* = 0.774), or ferritin (*p* = 0.318). Females had a mean Hb at T0 of 13.5 ± 1.3 g/dL (9.6; 16.1) and males had a mean of 15.0 ± 1.3 g/dL (11.7; 17.0). A total of 36 patients were randomized to the control group and 37 to the intervention group. After about 30 days of treatment (T1), the biochemical markers of martial status in the intervention group showed no statistically significant differences compared to the parameters in the control group. Details of means and medians of changes in blood values have been reported in Table 2.

We hypothesized that age could affect the response to treatment. We analyzed data considering the cut-off of 65 years and the percentage change from baseline of Hb (ΔHb). Of note, the younger group (*n* = 28) started with a mean Hb of 14.7 ± 1.3 g/dL (11.9; 17.0) and the older group (*n* = 45) started with a mean of 13.7 ± 1.4 g/dL (9.6; 16.6). Among older adults, the treatment variable was fairly correlated to ΔHb (*R* = 0.348; *p* = 0.019), with regression analyses revealing a positive linear association between age and response to treatment (*B* = 3.522; 95% CI: 0.604 to 6.441; *p* = 0.019).

**Figure 1:**
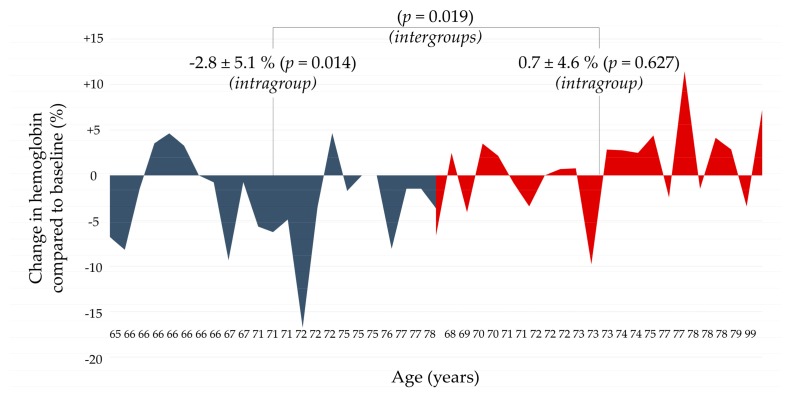
Area charts of hemoglobin changes, expressed as a percentage from baseline, in the subgroup of older adults within 30 days prior to hip or knee surgery. Control group: blue. Intervention group: red. The treatment consisted of daily supplementation of 30 mg of sucrosomial ferric pyrophosphate and 70 mg of L-ascorbic acid. In the *y*-axis is the percentage change in hemoglobin (%), whereas in the *x*-axis is the age of subjects. The oldest subjects in the control group appear to lose more hemoglobin than their youngest counterparts, but the oldest are also those that respond the more to treatment in the red group. In the control group, the cumulative absolute decrease in hemoglobin from baseline was −11.7 g/dL and in the intervention group was −4.6 g/dL.

Indeed, older adults who were randomized to the intervention group had a mean increase from baseline of 0.7 ± 4.6% (*p* = 0.627), compared to the control group that lost −2.8 ± 5.1% (*p* = 0.014) of baseline Hb. A statistically significant difference between groups was found in the levels of Hb at T1 (*p* = 0.019). In our cohort, patients over/equal 65 years showed to regularly lose significant points of Hb within 30 days prior to surgery (from 13.8 ± 1.1 to 13.4 ± 1.0 g/dL), with daily oral supplementation of 30 mg of sucrosomial ferric pyrophosphate and 70 mg of L-ascorbic acid being effective in prevent the loss (from 13.6 ± 1.7 to 13.7 ± 1.6 g/dL). The different response to treatment among older adults has been reported in Figure 1.

The treatment variable showed statistically significant effects also in the group of adults (*R* = −0.480; *p* = 0.010), with an inverse linear association (discrete, 0 = control, 1 = intervention; *B* = −4.736; 95% CI: −8.222 to −1.251; *p* = 0.010). Explicitly, adults who were treated had a mean decrease of −3.1 ± 5.0% (*p* = 0.024) and those in the control group had no differences (*p* = 0.194). This latter ambiguous effect was exploited after adjusting for gastrointestinal diseases and drugs that counteract the production of acid in the stomach. Within the whole study cohort, only three subjects reported to suffer from chronic intestinal disorders or had gastrointestinal surgeries (one gastric bypass, one chronic gastritis, and one gastroesophageal reflux disease (GERD)), and all were casually part of the treated group of adults. A fourth patient in the same subgroup used chronic antiacid medications, and they overall had no response to treatment. Excluding these four patients from the analysis, no more differences were observed in the group (*p* = 0.090). Of note, the other seven patients sparse within other subgroups reported to chronically use antiacids, but no differences in the results were observed after adjustments. In our cohort, control patients less than 65 years of age maintained serum Hb levels within 30 days prior to surgery. If adult patients suffered from gastrointestinal disease or chronically use antiacid medication, they might encounter a significant loss irrespective of concomitant oral supplementation with 30 mg of sucrosomial ferric pyrophosphate and 70 mg of L-ascorbic acid per day. Biochemical markers of martial status in the subgroup of older patients were reported in Table 3.

## 4. Discussion

In line with previous evidences [30], our cohort presented low rates of anemia (non-anemic patients: 91.8%) at preoperative anesthesia evaluation, but our eligibility criteria could have excluded the most complicated patients with martial status alterations. Patients were supplemented with an iron formula that had former evidence of efficacy [25], but—as previously described—even treatments supported by research evidence should be confirmed upon each new context of use. After 30 days of oral iron plus L-ascorbic acid therapy, no significant changes in the martial status were observed after treatment. The overall tolerability was 2.7% among treated patients (1 in 37). Although the subgroup of adults younger than 65 years of age did not respond to treatment and maintained their Hb levels approaching the day of surgery, older adults over/equal 65 years experienced an uncharacterized Hb loss. These significant reductions were averted upon treatment with iron formula. The martial status in adults appears to be more conservative, but diseases of the gastrointestinal system or past resection surgeries can expose patients to significant disturbances, which cannot be defused through standard iron supplementation. In the past, it was suggested that preoperative programs, such as exercise therapy [31], could be more meaningful and cost-effective if targeted to specific subpopulations. However, this may be not the case for dietary supplements. As a consequence, we choose a population medicine approach of “treating all instead of mass screening and then treating few” for the following reasons: I) Biochemical markers are not so sensitive and specific in discerning iron-deficiency anemia from other anemia types since also the subjects’ nutritional status and functional ability matter [9]. II) Normal baseline values may be rather irrelevant compared to the individual increase after iron therapy, which could provide more oxygenation to tissues even to healthy individuals who would have been excluded by treatment algorithms [20] because of normal Hb values (baseline Hb in older adults: 13.8 ± 1.1 g/dL). III) The set of rules for detecting, evaluating, and managing preoperative anemia might be unsuitable for high-performance centers, such as our IRCCS Orthopedic Institute Galeazzi that perform over 4000 interventions of major orthopedic surgery per year [9]. Screening all patients for martial and inflammatory status, vitamin profile, and the referral to gastroenterologists or nephrologists for individual treatment may be optimal, but not cost-effective.

Concerning the second reason, we undeniably took advantage in supplementing non-anemic older patients. In fact, among the control group of 23 older adults that encountered a significant reduction in Hb levels from baseline, over 95% resulted to be non-anemic at baseline, but lost a significant amount of Hb nevertheless. Using oral iron formulas in this group could have prevented the worsening of martial status. Older patients had a mean Hb at T0 that was lower than the younger group and this could certainly reflect lesser physiological reserves with lower ferritin deposits and higher demands for iron transport. Adults had ferritin levels of 121.5 ng/mL and Tf levels of 258 mg/dL, while older adults had 86.0 ng/mL and 253 mg/dL, respectively. We can hypothesize that our older subgroup could suffer a hybrid form of uncomplicated iron deficit with no acute inflammatory response [2]. Iron supply to erythropoiesis could have been becoming insufficient and Tf could have been upregulated to increase iron transport. Upon iron deficiency, first ferritin reduces and then Hb [32,33]. In this borderline condition, we may suppose an impairment of oxygen supply that could affect the functional ability nevertheless. The highest iron demands for erythropoiesis make a prolonged iron deficiency reflected by in low RBCs, and upon adequate iron integration, new erythrocytes take advantage of a positive iron pool (Table 3). Since it is a common thought that liver iron represents the main source upon bleeding, an increase in ferritin, reflecting higher iron deposits, would have been desirable. However, the higher median increase from the baseline of circulating μg of iron and Tf sat—even if not statistically significant—could have been enough to deliver more iron to the bone marrow and, in turn, augment RBC production.

We observed that gastrointestinal conditions, such as gastric bypass, gastritis, or GERD, cause adult patients to ineffectively absorb dietary iron. Other illnesses that certainly influence iron homeostasis are *Helicobacter pylori* infection and peptic ulcer disease [34]. Concerning medications, they are factors being part of the vast group of “iron absorption influencers”. These influencers can be disruptors (i.e., negative effectors) or enhancers (i.e., positive effectors), and are of great importance in the management of oral iron therapies: in fact, the metal easily changes its state of oxidation to form coordination complexes with other atoms capable of donating electrons, and absorption influencers can frustrate or potentiate the planned therapy. For instance, over 10% of our cohort patients reported to chronically use antacid medications, which are known to substantially reduce iron absorption. Other negative effectors on iron absorption are mainly of dietetic origin and can form insoluble salts in the stomach, such as oxalic and phytic acids, or compete for absorption through SLC11A2, such as manganese, zinc, and lead [35]. Furthermore, dietary calcium might inhibit the absorption of both heme and non-heme iron [36], whose interference is also experienced by plants [37], but even the food matrix itself might impede the availability [38]. Although we did not monitor the dietary intakes of disruptors or iron in our patients, which may represent a possible confounder in the interpretation of our results, future researchers may consider conducting dietary monitoring in their future studies. Indeed, recommended iron intakes for the lowest iron availability can even be set at 27.4 mg/die for men and 58.8 mg/die for women [39], considering the lowest observed adverse effect level being 70 mg/die [40]. In this study, we used a pharmaceutical formula that contained an absorption enhancer: the L-ascorbic acid. This water-soluble vitamin has a reducing potential able to prevent the oxidation of neighboring molecules. Despite only a small fraction of ingested vitamin C being absorbed, it is known to exert positive pharmaceutical actions in the lumen of the stomach and small intestine by reducing non-heme Fe^3+^ to Fe^2+^ and acting as a weak chelator, similarly to citric and lactic acid [41], to help solubilizing the metal [42]. In cells, L-ascorbic acid can promote the release of iron from deposits [43]. Of note, other positive effectors on iron absorption are fructose, copper [44], vitamin A, and β-carotene [45].

Poor dietary intake of bioavailable iron and L-ascorbic acid may aggravate martial status alterations in older adults undergoing elective orthopedic surgery. Even if there was no anemia condition, it would be particularly relevant to apply a population medicine approach to sustain each patient’s needs, and oral iron therapies are usually the first-line treatment for uncomplicated anemia. However, dietary supplements are often misused by patients [46,47], mainly because of their availability, ease of administration, and relatively low costs [48]. Dietary supplements do not lack in adverse effects [46,47], and intelligent interventions are uncommon and often non-adapted to age, sex, or lifestyle behaviors—such as inhabitation altitude or smoking habits [1]. The complexities of different pharmaceutical formulas to be integrated and the extent of absorption influencer interferences are often missed, possibly leading to ineffectiveness [49,50] or side effects [51,52,53]. The intravenous route may be chosen when patients do not tolerate oral administration or when the supplement has no effects, or when compliance to oral therapy is dubious [54]. In fact, the efficacy of oral formula in optimizing the martial status often requires daily administration whereas a single-dose of intravenous iron may be sufficient [55]. Our formula with iron plus L-ascorbic acid was effective in older adults, but perhaps more satisfying results would have been obtained if our patients had also been supplemented with cobalamin, folate, and vitamin A. It would also have been interesting to monitor more accurately iron markers, such as the concentrations of erythrocyte protoporphyrin (EP) and soluble transferrin receptors (sTfRs) [56], but also inflammatory parameters.

## 5. Conclusions

Humans rely on three mechanisms for iron incorporation: intestinal passage, ferritin allocation, and erythrocytes cycle. Despite these biological strategies, humans commonly suffer from iron deficiency syndromes. Poor oral iron intake and bioavailability, aging process, co-existing conditions, and absorption disruptors affect iron homeostasis. Literature evidences and laws recommendations stress the need to improve martial status before orthopedic surgery and this integration can be exploited in the orthopedic POC. However, no pragmatic connection exists between guideline statements and their effectiveness, and the identification of deficient patients relies on often-hardy applicable algorithms. We supported a population medicine approach, which has already been proved to be effective even in the most complicated patients [57], and obtained remarkable results dependent on age, gastrointestinal conditions, and medications. Instead of choosing patients that should or should not undergo oral iron supplementation, future POC may sustain a basic “iron prophylaxis” for all orthopedic patients. The concept of timing is extremely relevant, either within the same day or through alternative days, with important differences in tolerability and absorption [58,59]. Alternative solutions at the population level might be required in the near future to correct anemia long before surgery. For instance, the American National Heart, Lung, and Blood Institute defines healthy eating changes, and not oral iron supplementation, as first-line treatments for mild to moderate iron-deficiency anemia [60]. Managing a nutritional supplementation with iron sources is pertinent for the proper care of orthopedic patients that face important blood losses, with the key factor for success being always the same: a constructive multidisciplinary team capable to bring to light any relevant aspect to guarantee the entire orthopedic population a medicine as precise as possible [9,61].

## Figures and Tables

**Table 1:** Biochemical markers of martial status of study patients who were recruited about 30 days prior to hip or knee replacement during preoperative anesthesia evaluation.

	Randomization Step (Baseline, T0) (*n* = 73)		Prevalence out of Ref. Val. *n* (%)	
	♂ (*n* = 30)	♀ (*n* = 43)	♂	♀
Hb (g/dL)	15.0 ± 1.3 (11.7–17.0)	13.5 ± 1.3 (9.6–16.1)	2 (6.7)	3 (6.9)
[ref. val.]	[13.7–17.5]	[11.2–15.7]		
RBCs (10^6^/μL)	4.9 ± 0.4 (3.8–5.5)	4.8 ± 0.5 (3.8–6.1)	7 (23.4)	8 (18.6)
[ref. val.]	[4.63–6.08]	[3.93–5.22]		
MCV (fL/cell)	89.8 (88.2; 92.5)	88.4 (85.5; 90.8)	8 (26.7)	7 (16.3)
[ref. val.]	[79.0–92.2]	[79.4–94.8]		
MCH (pg/cell)	30.5 (29.7; 31.0)	29.0 (27.5; 30.2)	2 (6.7)	7 (16.3)
[ref. val.]	[25.7–32.2]	[25.6–32.2]		
MCHC (g/dL)	33.9 ± 0.9 (32.6–35.6)	32.7 ± 1.0 (30.7–34.8)	0 (0.0)	12 (27.9)
[ref. val.]	[32.3–36.5]	[32.2–35.5]		
Iron (μg/dL)	76.5 (65.5; 89.0)	67.0 (57.0; 83.5)	2 (6.7)	2 (4.6)
[ref. val.]	[31–144]	[25–156]		
Tf (mg/dL)	247.0 (227.3; 256.3)	268.0 (239.0; 296.0)	2 (6.7)	1 (2.3)
[ref. val.]	[163–344]	[180–382]		
Tf sat (%)	21.5 (18.3; 25.8)	17.0 (13.0; 23.0)	11 (36.7)	14 (32.6)
[ref. val.]	[20–50]	[15–50]		
Ferritin (ng/mL)	190.5 (125.5; 313.3)	75.0 (39.5; 125.0)	12 (40.0)	4 (9.3)
[ref. val.]	[22–275]	[5–204]		

Normally distributed values have been reported as means ± SD (min-max). Skewed values have been reported as medians (Q1; Q3). Abbreviations: ref. val. = reference values according to our laboratory, Hb = hemoglobin, RBCs = red blood cells, MCV = mean corpuscular volume, MCH = mean corpuscular hemoglobin, MCHC = mean corpuscular hemoglobin concentration, Tf = transferrin, Tf sat = transferrin saturation.

**Table 2:** Biochemical markers of martial status in patients randomized to control group (C) or treatment group (I) with 30 mg of sucrosomial ferric pyrophosphate and 70 mg of L-ascorbic acid daily during 30 days prior to hip or knee surgery.

		Randomization Step *(baseline, T0)*	Preoperative Step *(after 30 days, T1)*	*T0 p*-Values *	*T1 p*-Values *
Hb (g/dL)	C	14.3 ± 1.3 (11.7–16.4)	14.1 ± 1.2 (11.2–15.9)	*p* = 0.403	*p* = 0.259
	I	14.0 ± 1.7 (9.6–17.0)	13.8 ± 1.5 (10.7–16.6)		
RBCs (10^6^/μL)	C	4.9 ± 0.4 (3.8–5.7)	4.8 ± 0.4 (3.7–5.4)	*p* = 0.483	*p* = 0.584
	I	4.8 ± 0.6 (3.8–6.1)	4.7 ± 0.5 (3.6–6.0)		
MCV (fL/cell)	C	89.5 (86.3; 91.5)	88.1 (86.1; 92.3)	*p* = 0.608	*p* = 0.825
	I	89.0 (86.1; 92.0)	89.5 (85.3; 91.2)		
MCH (pg/cell)	C	30.0 (28.5; 30.6)	30.0 (28.5; 30.9)	*p* = 0.800	*p* = 0.627
	I	29.7 (28.5; 30.7)	29.7 (28.5; 30.9)		
MCHC (g/dL)	C	33.2 ± 1.0 (30.8–35.5)	33.5 ± 1.0 (31.9–35.6)	*p* = 0.904	*p* = 0.906
	I	33.2 ± 1.2 (30.7–35.6)	33.5 ± 1.4 (30.5–37.0)		
Iron (μg/dL)	C	74.0 (64.0; 85.0)	87.0 (66.0; 107.0)	*p* = 0.476	*p* = 0.830
	I	69.0 (61.0; 83.0)	94.0 (66.0; 110.0)		
Tf (mg/dL)	C	258.0 (234.8; 287.0)	256.5 (229.8; 291.8)	*p* = 0.402	*p* = 0.559
	I	253.0 (228.0; 285.0)	248.0 (229.0; 274.0)		
Tf sat (%)	C	20.0 (15.0; 23.0)	22.0 (16.8; 30.3)	*p* = 0.774	*p* = 0.515
	I	20.0 (16.0; 23.0)	24.0 (17.0; 32.0)		
Ferritin (ng/mL)	C	129.0 (75.0; 218.0)	151.5 (68.8; 229.0)	*p* = 0.318	*p* = 0.256
	I	100.0 (51.0; 181.0)	94.0 (51.0; 161.0)		

* The significant statistical difference between groups was calculated by using the independent sample *t*-test or the Mann–Whitney U test for normally distributed or skewed values, respectively. All tests were performed by using SPSS 22 and 2-tailed tests. Normally distributed values have been reported as means ± SD (min-max). Skewed values have been reported as medians (Q1; Q3). Abbreviations: Hb = hemoglobin, RBCs = red blood cells, MCV = mean corpuscular volume, MCH = mean corpuscular hemoglobin, MCHC = mean corpuscular hemoglobin concentration, Tf = transferrin, Tf sat = transferrin saturation.

**Table 3:** The percentage change from baseline of biochemical markers of martial status in patients over/equal 65 years old randomized to control group (C, *n* = 23) or treatment group (I, *n* = 22) with 30 mg of sucrosomial ferric pyrophosphate and 70 mg of L-ascorbic acid daily during 30 days prior to hip or knee surgery.

	30-Days Changes (T1-T0)%		*p*-Values *
	*C*	*I*	
Δ *Hb	−2.8 ± 5.1	0.7 ± 4.6	*p* = 0.019
ΔRBCs	−3.3 ± 5.7	0.0 ± 4.8	*p* = 0.041
ΔMCV	−0.2	−0.1	*p* = 0.923
ΔMCH	0.3	0.0	*p* = 0.708
ΔMCHC	0.8 ± 2.0	1.1 ± 4.5	*p* = 0.806
ΔIron	24.2	40.8	*p* = 0.709
ΔTf	−0.2	0.9	*p* = 0.904
ΔTIBC	20.0	42.6	*p* = 0.754
ΔFerritin	7.3	1.7	*p* = 0.952

* Data has been reported as the mean percentage change from baseline values. ** The significant statistical difference between groups was calculated by using the independent sample *t*-test or the Mann–Whitney U test for normally distributed or skewed values, respectively. All tests were performed by using SPSS 22 and 2-tailed tests. Abbreviations: Hb = hemoglobin, RBCs = red blood cells, MCV = mean corpuscular volume, MCH = mean corpuscular hemoglobin, MCHC = mean corpuscular hemoglobin concentration, Tf = transferrin, Tf sat = transferrin saturation.

**Figure A1:**
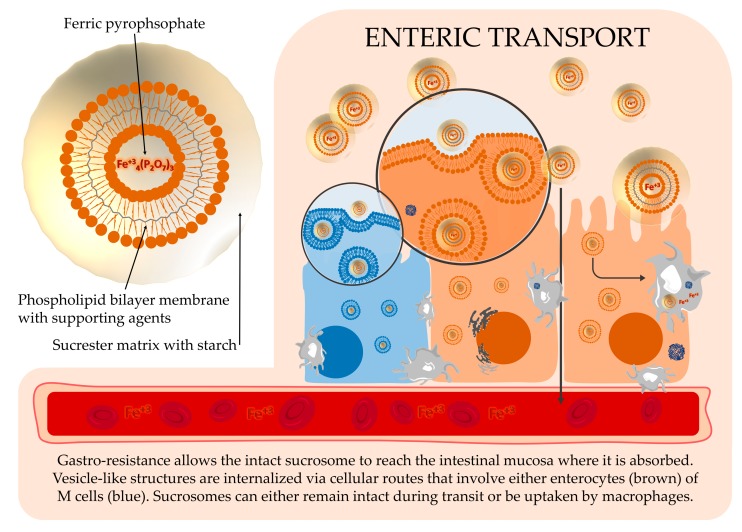
Schematics of the formula that was used in the research study and the suggested mechanisms of intestinal absorption. Absorption starts with passive transport by enterocytes or specialized epithelial cells of the mucosa-associated lymphoid tissues (M cells), and concludes with macrophage degradation and storage.

## Supplementary Materials

Supplementary File 1

## Appendix A. Dietary Supplement

Our study patients were supplemented with a daily capsule containing 30 mg of iron in the ferric pyrophosphate form surrounded by a sucrosomial matrix made of phospholipids and sucrester (SiderAl Forte, PharmaNutra). The iron coordination entity is composed of Fe^3+^ cations and diphosphate^4-^ anions in a 4:3 ratio. The sucrosomial matrix is stabilized by tricalcium phosphate bindings that reduce lipid mobility [62], with other ingredients being incorporated, such as a pre-gelatinized rice starch, glucose syrup, and milk proteins, collectively forming the “sucrosome”. In particular, the sucrester is a surfactant derived from the esterification of fatty acids with sucrose (sucrose esters) and it allows the formula to be gastro-resistant and to be highly absorbed in intestines [63]. The dietary supplement that was used in this research also contained different components classified as bulking agents (microcrystalline cellulose, edible gelatine), anti-oxidant (L-ascorbic acid), anti-caking agents (hydroxypropyl methylcellulose, magnesium salts of fatty acids, silicon dioxide), and coloring agent (titanium dioxide). The increased absorption of this formula was reported to facilitate the enteric transport through paracellular and transcellular routes [64]. In particular, it was shown that the sucrosome is mostly absorbed as a vesicle-like structure through endocytosis-mediated cellular uptake, bypassing the conventional iron absorption pathway [65]. Immune cells may be involved in the iron acquisition from sucrosomes. In fact, resident macrophages in the intestines, which are known to stabilize the enteric barrier function through intraepithelial projections [66], could incorporate the sucrosome, obtain iron, and subsequently release it into the plasma when needed. Indeed, red pulp macrophages are known to be involved in the clearance of aged RBCs and iron recycling. These resident immune cells in the gut may even act as transient iron deposits [65]. M cells of Peyer’s patches, which comprise absorptive cells and hollows that enfold defensive components, may also have a role in sucrosome absorption. In fact, these cells usually transfer microbes or other particles from the luminal face to the *lamina propria* where are resident immune cells, and their mediated pathway for sucrosome absorption could ultimately involve the taking up by macrophages [63], thus fulfilling the abovementioned roles.

## Appendix B

**Table A1:** Blood-based indicators of martial status.

Marker	Description	Ref. val.	Significance
Hb	Assembly of four globular polypeptide chain, with each one being associated with a prosthetic heme group that contains an atom of iron either in the ferrous or in the ferric state. The four oxidized ion atoms in Hb carry four oxygen molecules.	♂ [13.7–17.5 g/dL] ♀ [11.2–15.7 g/dL]	Hb concentration in blood is a measure for anemia. Low serum values reflect low functional iron when there are no concurrent infective/inflammatory disorders or other micronutrient deficits, such as vitamin A or B group.
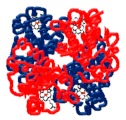
RBCs	Anucleated biconcave disks with an aphospholipid bilayer. They lack most organelles and appear with a central pallor and surrounding warp filled with Hb. The deformable shape allows the traversing of the smallest capillaries.	♂ [4.63–6.08 10^6^/μL] ♀ [3.93–5.22 10^6^/μL]	They reflect the intensity of erythropoiesis when there are no concurrent B vitamin deficiencies, or diseases of kidneys, liver, and thyroid. Low RBCs can mirror an iron depletion.
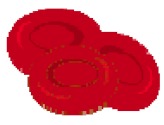
MCV	Average size/volume of a red blood cell. Calculated as the ratio of hematocrit, which measures the volume percentage of RBCs, to RBCs concentration. The higher the MCV the greater the average size/volume of erythrocytes.	♂ [79.0–92.2 fL/cell] ♀ [79.4–94.8 fL/cell]	Indicative of a correct erythropoiesis. When bone marrow lacks a proper iron supply, MCV is low and RBCs are microcytic. A deficiency of cobalamin or folate results in higher MCV, which is a condition named macrocytic anemia.
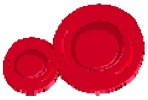
MCH	Indirect index calculated as the ratio of Hb to RBCs. Haemoglobin molecules in erythrocytes are located in the periphery and surround a central pallor. The more extensive the central pallor the lesser Hb is contained (left erythrocyte).	♂ [25.7–32.2 pg/cell] ♀ [25.6–32.2 pg/cell]	MCH value closely parallels the value of MCV. Defects in nuclear maturation, such as in megaloblastic anemia, result in high values of MCH.
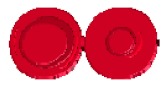
MCHC	Indirect index calculated as the ratio of Hb to the volume percentage of RBCs. The value of MCHC is increased in spherocytosis as erythrocyte assume a spherical shape because of the loss of membrane (erythrocyte on the right).	♂ [32.3–36.5 g/dL] ♀ [32.2–35.5 g/dL]	It correlates Hb with RBCs volume. Low values can reflect micronutrient deficiencies. When no genetic disease or hemolysis is present, high values are mostly artefact (lipemia), because RBCs cannot contain more Hb than normal.
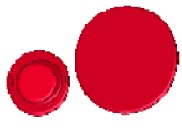
Iron	The amount of the metal that is circulating in blood, primarily bound to proteins, such as Tf and ferritin. A slightest part is non-transferrin bound iron (NTBI) and has capacity to generate highly reactive free radicals.	♂ [31–144 μg/dL] ♀ [25–156 μg/dL]	The highest serum levels can result from intravenous iron or genetic diseases, whereas lowest concentrations can be found in anemias for inflammation or chronic diseases.
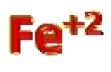
Tf	A single polypeptide chain and two carbohydrate chains forms Tf. When no iron is bound, the protein is called apotransferrin. Tf binds a maximum of two atoms of ferric iron for its solubilisation and reactiveness reduction.	♂ [163–344 mg/dL] ♀ [180–382 mg/dL]	It transports iron and reflects, similarly to its receptors on RBCS, the demand of iron. If there are no other reasons for abnormal erythropoiesis, its serum levels increase when iron stores are exhausting. It is a negative acute-phase protein.
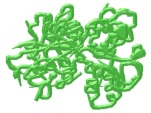
Tf saturation	The binding sites on all Tf molecules occupied with iron. It is calculated as the ratio of serum iron to Tf or serum iron to total iron binding capacity (TIBC), the latter being the total amount of blood iron that can be bound by proteins.	♂ [20–50%] ♀ [15–50%]	It provides information, similarly to Tf, about the adequacy of iron supply to meet cellular requirements. High degrees of saturation identify patients at risk of iron overload.
Ferritin	A 24-units globular protein with both light and the heavy chains that takes up to 4300 iron atoms to be deposited in its core of few nm. The form of iron deposit consists of crystals of ferric hydroxides and phosphates.	♂ [22–275 ng/mL] ♀ [5–204 ng/mL]	Its serum levels represent a small fraction of the body’s ferritin pool. Low serum values reflect a depletion of iron stores when there are no concurrent infections or vitamin C deficits. It is a positive acute-phase protein.
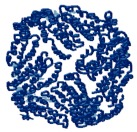

Abbreviations: Ref. val. = reference values for males and females, Hb = hemoglobin, RBCs = red blood cells, MCV = mean corpuscular volume, MCH = mean corpuscular hemoglobin, MCHC = mean corpuscular hemoglobin concentration, Tf = transferrin, Tf sat = transferrin saturation.
